# SiPM-MAROC gamma-camera prototype with monolithic NaI(Tl) scintillator

**DOI:** 10.1186/2197-7364-2-S1-A49

**Published:** 2015-05-18

**Authors:** Dmitry Philippov, Andrey Ilyin, Vladimir Belyaev, Elan Popova, Pavel Buzhan, Alexei Stifutkin

**Affiliations:** National Research Nuclear University, Moscow Engineering Physics Institute, Russia; Moscow Engineering Physics Institute, Russia

A full-body gamma-camera based on SiPM readout is currently under development as a part of MEPHI R activity supported in the framework of Russian Megagrants program. A goal of this development is a fast upgrade of existing medical equipment with minor changes in a system design and construction in order to combine SPECT and MR instruments. A monolithic NaI(Tl) scintillator commonly used for conventional PMT-based gamma cameras has been chosen for this study. SiPMs will be coupled with the scintillator via an optical guide. To cover scintillator surface thousands of SiPMs are required, together with multichannel front-end electronics. That means that readout electronics have to be very compact, with low power consumption and low cost. 64 – channel ASIC MAROC from Weeroc provides individual readout of each SiPM and has been considered as the best choice among electronics solutions available on the market. As the photodetector parameters are the key issues here, KETEK SiPMs with high detection efficiency, low crosstalk and low noise have been chosen for this study. In order to study the proposed detection system in detail and obtain detector module parameters, required for MC simulation, a 64-channel small prototype with 6x6mm^2^ SiPMs has been constructed and tested. SiPMs in SMD packages have been assembled as a matrix of 8x8 elements and readout by MAROC-based board. Prototype has been tested with different shape NaI(Tl) scintillators and gammas with different energy. Dedicated algorithms for extraction of gamma-event’s energy and position are under development. They are based on fitting a matrix of individual SiPMs responses by an analytical function F(x,y). They will be tested with GEANT-simulated events and experimental data. Development of the next (engineering) prototype of SiPM’s module for gamma-camera will be started soon.

